# Beneficial Effects of Total Phenylethanoid Glycoside Fraction Isolated from *Cistanche deserticola* on Bone Microstructure in Ovariectomized Rats

**DOI:** 10.1155/2019/2370862

**Published:** 2019-06-27

**Authors:** Lingling Yang, Shuqin Ding, Bo Zhang, Jingjing Liu, Yanhong Dong, Qiwen Tang, Pingping Yang, Xueqin Ma

**Affiliations:** ^1^Department of Pharmaceutical Analysis, School of Pharmacy, Ningxia Medical University, 1160 Shenli Street, Yinchuan 750004, China; ^2^School of Clinical Medicine, Ningxia Medical University, 692 Shenli Street, Yinchuan 750004, China; ^3^Key Laboratory of Hui Ethnic Medicine Modernization, Ministry of Education, Ningxia Medical University, 1160 Shenli Street, Yinchuan 750004, China

## Abstract

The present study was designed to estimate the antiosteoporotic activity of total phenylethanoid glycoside fraction isolated from *C. deserticola* (CDP) on rats induced by ovariectomy (OVX) as well as the related mechanisms. After 3 months of oral administration, the decreased bone mineral density, serum Ca, and P in OVX rats were recovered and the deteriorated trabecular bone microarchitecture was partly improved by CDP (60, 120, and 240 mg/kg) intervention, the activities of bone resorption markers were downregulated, and the bioactive of the bone formation index was upregulated; meanwhile, the content of MDA was declined, and GSH was increased by CDP treatment. Compositionally, 8 phenylethanoid glycoside compounds were identified in CDP, with the total contents quantified as 50.3% by using the HPLC method. Mechanistically, CDP declined the levels of TRAF6, RANKL, and RANK, thus suppressing RANKL/RANK/TRAF6-induced activation of downstream NF-*κ*B and PI3K/AKT signaling pathways and ultimately preventing activities of the key osteoclastogenic proteins of NFAT2 and c-Fos. All of the above data implied that CDP exhibited beneficial effects on bone microstructure in ovariectomized rats, and these effects may be related to the NF-*κ*B and PI3K/AKT signaling pathways which were triggered by the binding of RANKL, RANK, and TRAF6.

## 1. Introduction

Postmenopausal osteoporosis, where 1 in 3 women older than 50 years will suffer, is becoming a main health hazard afflicting more than 200 million women all over the world [1]. At menopause, the sharp decline of the estrogen level usually leads to an exceed bone resorption caused by enhanced osteoclastogenesis; then, the balance between osteoblast-induced bone formation and osteoclast-induced bone resorption was disrupted, and the accelerated bone resorption finally caused osteoporosis and even hip or spine fracture [2]. It was believed that the differentiation of the osteoclast was triggered when receptor activator of nuclear factor kappa B (RANK) bound to RANKL, the ligand of RANK. However, the combination of RANK to RANKL cannot be activated unless protein tumor necrosis factor receptor-associated factor 6 (TRAF6) was joined in it [3], followed by the stimulation of downstream signaling pathways including PI3K/AKT and NF-*κ*B. And finally, the expressions of nuclear factor of activated T cells c2 (NFAT2) and c-Fos were regulated [4] to modulate the differentiation of the osteoclast as well as bone resorption. Thus, the factors and regulators which are directly or indirectly related to the activation and differentiation of osteoclast were believed as crucial targets for preventing bone loss.

There are indeed some clinical and synthetic hormone replacement therapy drugs like estradiol valerate which is effective on treatment of postmenopausal osteoporosis. Unfortunately, some of which enhanced the risk of serious cancers including breast and endometrial cancers [5], which limited their clinical applications. Therefore, it is necessary to select other alternatives with both efficacy and minimal side effects. Traditional Chinese medicines (TCM), as well as the isolated bioactive compounds and fractions [6–9], were proved effective on various ailments including postmenopausal osteoporosis. Among these bioactive components and fractions, phenylethanoid glycoside (PhG) compounds with potential efficacy were believed as promising agents for the treatment of osteoporosis [10–12]. The structures of PhGs consist of cinnamic acid aglycone, a hydroxyl phenyl ethyl group which is combined with *β*-glucopyranose, apiose, galactose, rhamnose, or xylose via a glycosidic bond. They widely exist in medicinal species of genus *Cistanche* [13]. *Cistanche deserticola* Y.C. Ma is an official TCM which is recorded in Chinese pharmacopoeia, and besides being an important TCM [14], *C deserticola* is also an antiaging tonic herb with few side effects which has been developed into medicinal liquor and nutritional liquid approved by the State Food and Drug Administration. Based on the record of Chinese pharmacopoeia, *C*. *deserticola* had been traditionally used by natives to handle kidney essence deficiency problems like muscle debility and lumbar weakness, and phenylethanoid glycoside compounds including echinacoside and acteoside are the main bioactive constituents in this herb. According to the TCM theory of “kidney-govern-bone,” the bone system is governed by kidney essence [15], and the bone-related troubles like osteoporosis could be recovered by herbs or compounds possessing the activity of nourishing the kidney essence. Therefore, we hypothesized that the total phenylethanoid glycoside fraction isolated from *C*. *deserticola*, at least partly, was beneficial on the treatment of osteoporosis. The current experiment was therefore devised to validate our hypothesis by using an ovariectomized (OVX) rat model; besides the bone resorption and formation markers which must be estimated, the antioxidation index as well as RANKL/RANK/TRAF6-induced PI3K/AKT and NF-*κ*B signaling pathways were also employed to investigate the main mechanisms of the antiosteoporotic bioactivity.

## 2. Materials and Method

### 2.1. Plant Materials and Preparation

A total of 30 kg stems of *Cistanche deserticola* Y.C. Ma were collected from Yongning County in September of 2015 with the coordinates 106.026597 and 38.262816, Ningxia Province, China. The herb was identified by Dr. Lin Dong (Department of Pharmacognosy, Ningxia Medical University), and a corresponding specimen (#20150901) was preserved in the Department of Pharmaceutical Analysis. Firstly, 30.0 kg of powdered *C. deserticola* was extracted by using the reflux method with 70% ethanol as solvent; the ratio of material to solvent was set as 1 : 10, and the reflux time was 2 h for 3 times. Then, all of the filtrates were combined together and condensed under reduced pressure at 60°C. Secondly, AB-8 macroporous resin columns were used for the preliminary separation, and different ratios of ethanol in water (0%, 20%, 30%, 40%, 50%, and 60%, each 60 L) were employed for eluting. Thirdly, the 40% and 50% eluents were combined and further purified by using repeated AB-8 macroporous resin columns with the eluents of 0%, 20%, 30%, 40%, and 50% ethanol in water, and each eluent was 12 L. Finally, the 40% fraction was collected and condensed under reduced pressure to obtain 150 g pale yellow sediment phenylethanoid glycoside fraction of *C. deserticola* (CDP, the yield was 0.5%). For *in vivo* experiments, 0.5% CMC-Na solvent was employed to dissolve CDP; oral administration to animals was set as 1 mL/100 g of body weight; for *in vitro* Western blot analysis, CDP was dissolved with DMSO and then diluted with DMEM to obtain the final concentrations of 0.1 mg/mL, 0.01 mg/mL, and 0.001 mg/mL.

### 2.2. Chemicals and Solvents

Estradiol valerate (EV) was from Delpharm Lille S.A.S., France; alkaline phosphatase (ALP), bone gla-protein (BGP), tartrate-resistant acid phosphatase (TRAP), and deoxypyridinoline (DPD) crosslink ELISA kits from Xinyu Biological Engineering Co. Ltd., Shanghai, China, 201605; malondialdehyde (MDA, 20181221), superoxide dismutase (SOD, 20121218), and glutathione (GSH, 20181221) reagent kits from Institute of Nanjing Jiancheng Biological Engineering, Nanjing, China; lntact parathormone (l-PTH, NEWASHE7UZ), calcitonin (2L9ISN7AIU), and estrogen-related receptor alpha (ERR*α*, Y3AY8QEWB3) crosslink ELISA kits from Elabscience Biotechnology Co. Ltd., Wuhan, China; cathepsin K ELISA reagent kit from BioVision, America, 1l300141; primary antibodies of RANKL (GR3193842-5), RANK (AA02113656), TRAF6 (2), c-Fos (AG12059411), NFAT2 (AO11015648), NF-*κ*B-p65 (AH04138226), PI3 kinase p85 alpha (AC09021266), AKT 1 (AF05173234), *β*-actin (17AV0411), and secondary antibodies of horseradish peroxidase-conjugated goat anti-rabbit IgG from ZSGB-BIO, China, 136080; total BCA protein assay kit and the commercial kit for the detection of osteoclast formation and fetal bovine serum and Dulbecco's modified Eagle's medium (DMEM) from HyClone, Logan, UT, USA; polyvinylidene fluoride (PVDF) membrane from Millipore Life Sciences, Billerica, MA, USA; penicillin and streptomycin from Gibco, Rockville, MD, USA. All the other chemical agents used were of AR grade.

### 2.3. HPLC Quantification of CDP

**Figure 1:**
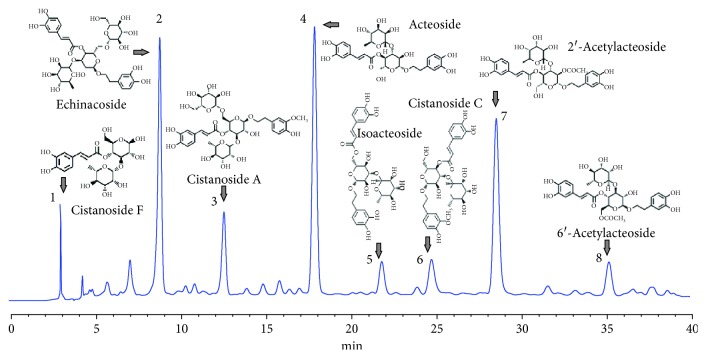
HPLC fingerprint of CDP. Eight phenylethanoid glycoside compounds were found in this fraction, and the total contents were quantified as 50.7%. The compounds and their contents were as follows: (1) acteoside F (3.6%), (2) echinacoside (8.8%), (3) cistanoside A (5.0%), (4) acteoside (13.3%), (5) isoacteoside (3.3%), (6) acteoside C (3.6%), (7) 2′-acetylacteoside (9.9%), and (8) 6′-acetylacteoside (3.2%).

An Agilent 1220 HPLC instrument was employed to identify and quantify the composition of CDP. The chromatography conditions were as follows: C18 column (TSK-GEL, 4.6 i.d.×250 mm, 5 *μ*m); gradient elution contained solvents A (acetonitrile) and B (water containing 0.5% acetic acid) (0-10 min: 17-20% A; 10-30 min: 20-25% A; and 30-40 min: 25-30% A); the detection wavelength was 333 nm; ambient temperature; flow rate was 1.0 mL/min; sample injection volume was 5 *μ*L. Eight PhG compounds, namely, cistanoside F, echinacoside, 6′-acetylacteoside, cistanoside C, cistanoside A, acteoside, 2′-acetylacteoside, and isoacteoside, were identified; by using the corresponding reference substances and an external standard method, the contents of the above 8 PhGs were quantified by HPLC analysis (Figure 1).

### 2.4. Animal Experimental Protocol

A total of 60 female adult Sprague-Dawley rats aged 3 months were purposed from the center of animal testing of Ningxia Medical University, with the average initial body weights of about 234 ± 25 g. The rats were housed in a standard specific pathogen-free environment to acclimate for 1 week. Then, all of the rats were anesthetized (chloral hydrate, 100 mg/kg, i.p.) only or sham ovariectomized (SHAM), or two ovaries were both removed and then randomly divided into 5 subgroups: orally treated with vehicle (0.5% CMC-Na) was set as the model group (OVX), estradiol valerate (1 mg/kg/day) as the positive group (EV), and 60, 120, and 240 mg/kg/day of CDP as low (CDPL), moderate (CDPM), and high (CDPH) dosage groups, respectively. All the rats were orally administered daily and lasted for 3 months with the dosage adjusted every 2 weeks which depended on the change of the whole body weights. At the last day of the animal experiment, 24-hour urine was obtained by using metabolic cages; serum was collected from the femoral artery of anesthetized rats; the right femora, tibia, and all the organs were dissected and stored at -80°C for further analysis. The animal experiments that we conducted were approved by the Institutional Animal Care and Use Committee of Ningxia Medical University.

### 2.5. Bone Mineral Density Determination and Micro-CT Analysis

Firstly, a dual-energy X-ray absorptiometry machine (Lunar, USA) was used to estimate the total bone mineral density of the right femur of each rat; secondly, the same femur was used to estimate the 3D image of trabecular bone microarchitecture by employing a micro-CT scanner apparatus (GE, America). The isotropic resolution was set as 14 *μ*m to obtain an ideal 3D image; the region of interest (ROI) was chosen by setting the same coordinates in the growth plate of the femur of each sample; and the bone morphometric parameters including trabecular separation (Tb.Sp), trabecular number (Tb.N), trabecular thickness (Tb.Th), bone mineral content (BMC), tissue mineral density (TMD), and tissue mineral content (TMC) were obtained by analyzing the ROI.

### 2.6. Serum and Urine Biochemical Assay

The activities of serum cathepsin K, TRAP, SOD, and GSH as well as the contents of serum PTH, calcitonin, ERR*α*, MDA, BGP, and urine DPD were determined by employing corresponding reagent kits according to the manufacturer's instruction, and the level of alkaline phosphatase (ALP) and the contents of serum and urine calcium (Ca) and phosphorus (P) were estimated by employing an automatic machine (Ciba-Corning 550 Diagnostics Corp., Oberlin, OH, USA).

### 2.7. Western Blot Analysis

Osteoclasts were induced by using RAW 264.7 cells added with macrophage colony-stimulating factor (MCSF) and RANKL. Briefly, 1 × 10^7^ RAW 264.7 cells were cultured in a 6-well plate in the presence of 30 ng/mL of MCSF and 25 ng/mL of RANKL. After 6 days of induction, the matured osteoclast cells were identified by using the TRAP-stained method with the corresponding kit, then treated with CDP (0.1, 0.01, and 0.001 mg/mL, respectively) for 24 h; then, the cells were lysed with a lysis buffer containing 0.5 mmol phenylmethylsulfonyl fluoride, protease and phosphatase inhibitors. The lysate was then separated by using 10% SDS-PAGE and transferred to a PVDF membrane, which was probed with AKT1, NF-*κ*B-p65, RANKL, PI3K-p85*α*, RANK, NFAT2, TRAF6, c-Fos, and *β*-actin (1 : 400) after blocking with 5% nonfat milk for 2 h. The same membranes were stripped and probed again with the above 9 corresponding antibodies, respectively, then were detected by the Image Lab software at the end. The experiments were repeated three times.

### 2.8. Statistical Analysis

All of the data obtained from *in vivo* and *in vitro* experiments, described as the mean ± SD, were analyzed by using one-way ANOVA followed by Dunnett's test (SPSS 22.0 software, SPSS, USA); *p* < 0.05 was statistically significant.

## 3. Results

### 3.1. Chemical Composition of CDP

By using the HPLC method, eight phenylethanoid glycoside compounds were found in this fraction, as Figure 1 show. By using standard references and an external standard method, the compounds and their contents were identified and quantified as follows: (1) acteoside F (3.6%), (2) echinacoside (8.8%), (3) cistanoside A (5.0%), (4) acteoside (13.3%), (5) isoacteoside (3.3%), (6) acteoside C (3.6%), (7) 2′-acetylacteoside (9.9%), and (8) 6′-acetylacteoside (3.2%). The total contents of these eight components were quantified as 50.7%.

### 3.2. Effects of CDP on Bone Mineral Density and Microarchitecture of Trabecula

**Figure 2:**
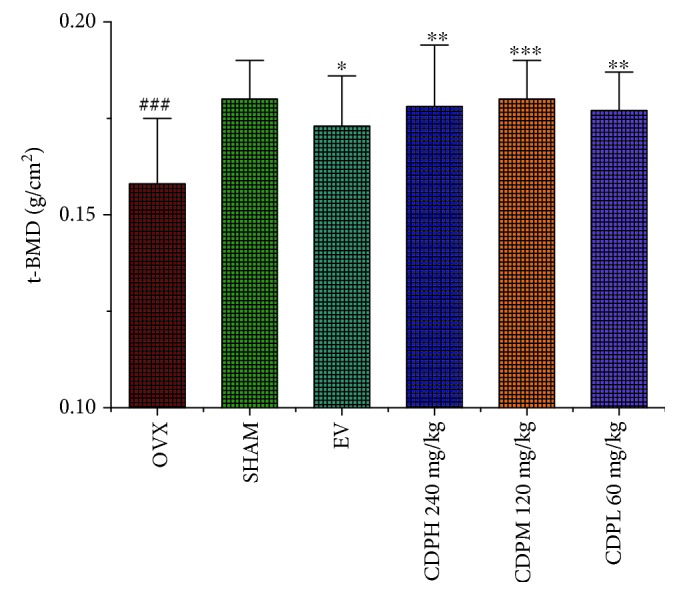
Effects of OVX and 12 weeks of treatment with CDP or EV on total bone mineral density in the right femur of rats which are assessed by using dual-energy X-ray absorptiometry (*n* = 10/group). Data were presented as the mean ± SD; ^∗^*p* < 0.05, ^∗∗^*p* < 0.01, and ^∗∗∗^*p* < 0.001 versus the OVX group; ^###^*p* < 0.001 versus the SHAM group.

**Figure 3:**
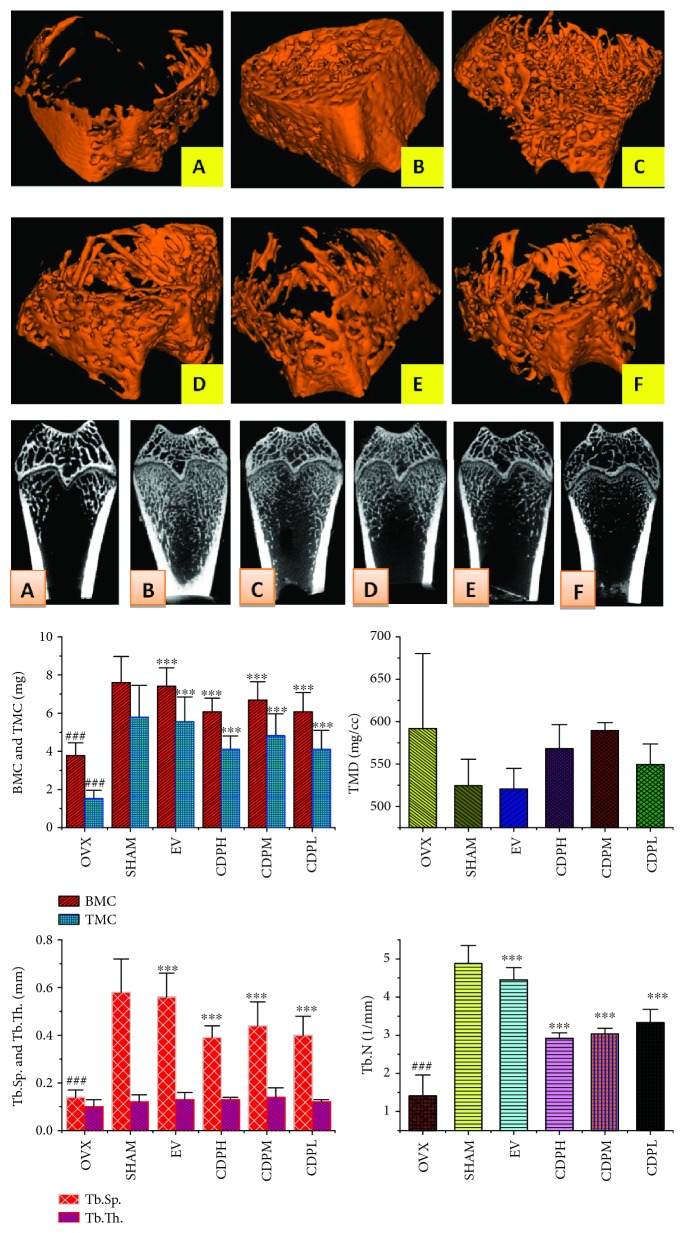
Micro-CT scan images of microarchitecture of the right femur of CDP-treated rats; the photographs shown were representative of 4 different rats in each group: (A) OVX group; (B) SHAM group; (C) EV group; (D) CDPH group; (E) CDPM group; (F) CDPL group. The measured parameters include bone mineral content (BMC), tissue mineral content (TMC), tissue mineral density (TMD), trabecular separation (Tb.Sp), trabecular number (Tb.N), and trabecular thickness (Tb.Th). The OVX rats expressed notable reduction of the microarchitecture area and trabecular number. CDP-treated rats and EV-treated rats partly reversed the abovementioned findings at the same degree after 12 weeks of treatment. All values were presented as the mean ± SD. ^∗^*p* < 0.05, ^∗∗^*p* < 0.01, and ^∗∗∗^*p* < 0.001 versus the OVX group; *^###^p* < 0.001 versus the SHAM group.

The total bone mineral density of the rats in different subgroups was shown in Figure 2. An obviously decreasing trend in the content of bone mineral density was observed in rats of the OVX model group, which decreased by nearly 12.0% after 12 weeks of the operation as compared with the rats of the SHAM group (*p* < 0.001). All of CDP-treated rats exhibited significantly increased bone mineral density by 11.2%, 12.0%, and 10.7% (*p* < 0.01), respectively, as compared with the rats of the OVX model group. Furthermore, consistent with the data of total bone mineral density, micro-CT reconstruction as well as histomorphometric determination of the femur showed that the rats in the OVX group showed obvious deterioration in trabecular architecture evidenced by the notably declined number and area of trabecula as well as markedly increased Tb.Sp when compared with the rats of the SHAM group. Treatment with CDP prevented the OVX-induced deterioration in trabecular architecture; as Figure 3 show, the BMC, TMC, and Tb.N values were significantly increased and the area of Tb.Sp was notably decreased, while the values of TMD and Tb.Th were seemed not significantly affected by the OVX operation and our CDP intervention.

### 3.3. Effects of CDP on Urine and Serum Biochemical Parameters

**Figure 4:**
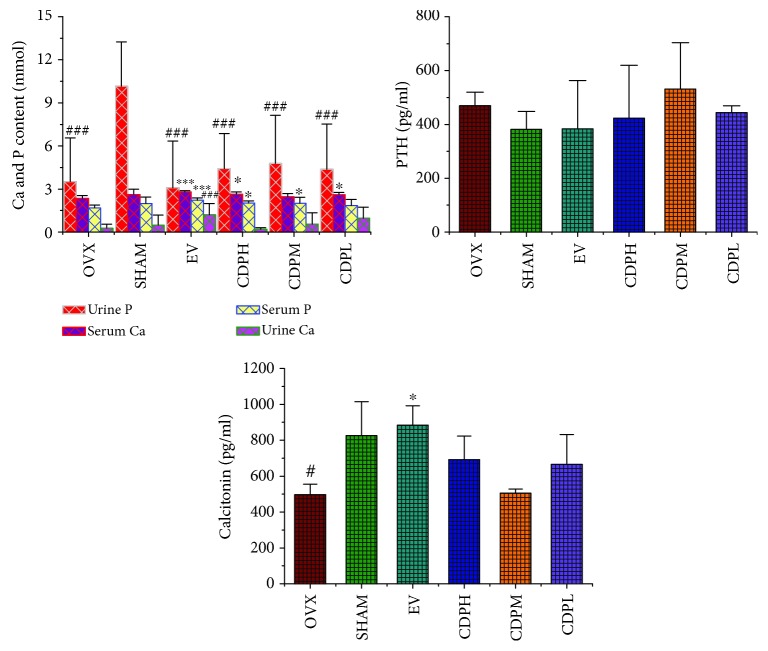
Effects of OVX and 12 weeks of treatment with CDP or EV on urine and serum Ca and P as well as PTH and calcitonin of rats (*n* = 10/group). All data were expressed as the mean ± SD. ^∗^*p* < 0.05, ^∗∗^*p* < 0.01, and ^∗∗∗^*p* < 0.001 versus the OVX group; ^#^*p* < 0.05, ^##^*p* < 0.01, and ^###^*p* < 0.001 versus the SHAM group.

As Figure 4 show, significant decrease trends of the urinary level of P and serum content of calcitonin in rats of the OVX model group were detected, which was nearly 30% and 60% less than the SHAM rats (*p* < 0.001), respectively, whereas no obvious increasing or decreasing trends in urinary levels of Ca and serum Ca as well as serum P and serum PTH were observed between OVX and SHAM groups. Treatment with CDP significantly prevented the loss of the serum P and Ca in OVX rats, evidenced by the levels of serum P and Ca notably upregulated (*p* < 0.05) as compared to the rats of the OVX model group. In addition, increased but nonstatistically significant trends of calcitonin were observed in both the low and high dosage groups of CDP as compared to the OVX model group.

### 3.4. Effects of CDP on Bone Formation and Bone Resorption Markers

**Figure 5:**
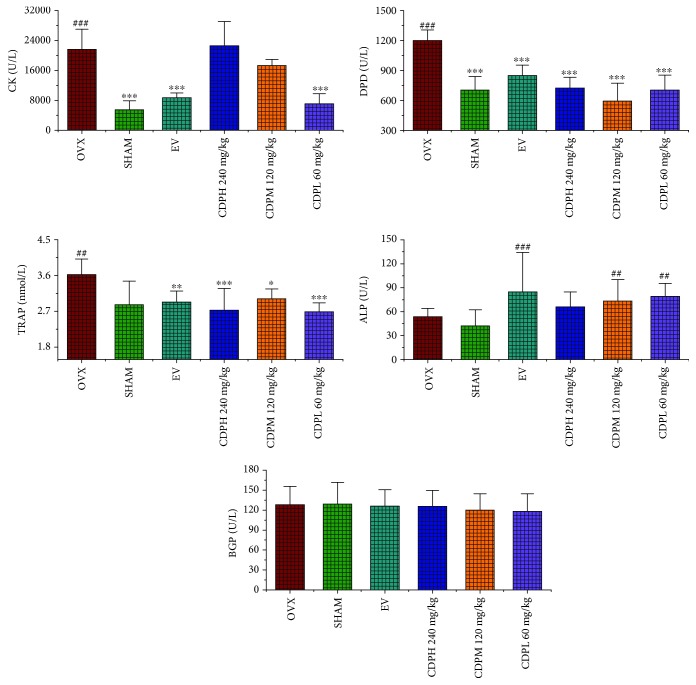
Effects of OVX and 12-week treatment with CDP or EV on serum TRAP, cathepsin K, DPD, ALP, and BGP activities of OVX rats (*n* = 10/group). All values were presented as the mean ± SD. ^∗^*p* < 0.05, ^∗∗^*p* < 0.01, and ^∗∗∗^*p* < 0.001 versus the OVX group; ^#^*p* < 0.05, ^##^*p* < 0.01, and ^###^*p* < 0.001 versus the SHAM group.

The beneficial effects of CDP on the bone formation index as well as inhibition influences on bone resorption markers were described in Figure 5. Concerning the bone formation markers, the levels of serum BGP were almost not influenced by the ovariectomized surgery evidenced by nonsignificant changes observed in all treated groups, whereas statistically significant improvements of serum ALP were obtained both in low (60 mg/kg) and moderate (120 mg/kg) dosages of CDP intervention groups when compared with the rats of the SHAM group (*p* < 0.01). Concerning the bone resorption index, the levels of serum cathepsin K and DPD as well as TRAP in rats of the OVX model group were significantly enhanced by about 75.0%, 41.4%, and 21.0%, respectively, as compared with the SHAM rats, and when treated with CDP, especially the low dosage of 60 mg/kg, the properties of cathepsin K and DPD as well as TRAP in the OVX model group were notably inhibited by 67.3%, 41.4%, and 25.9%, respectively, as compared to the rats in the OVX model group.

### 3.5. Effects of CDP on the Vagina and Uterine as well as Whole Body Weights

**Figure 6:**
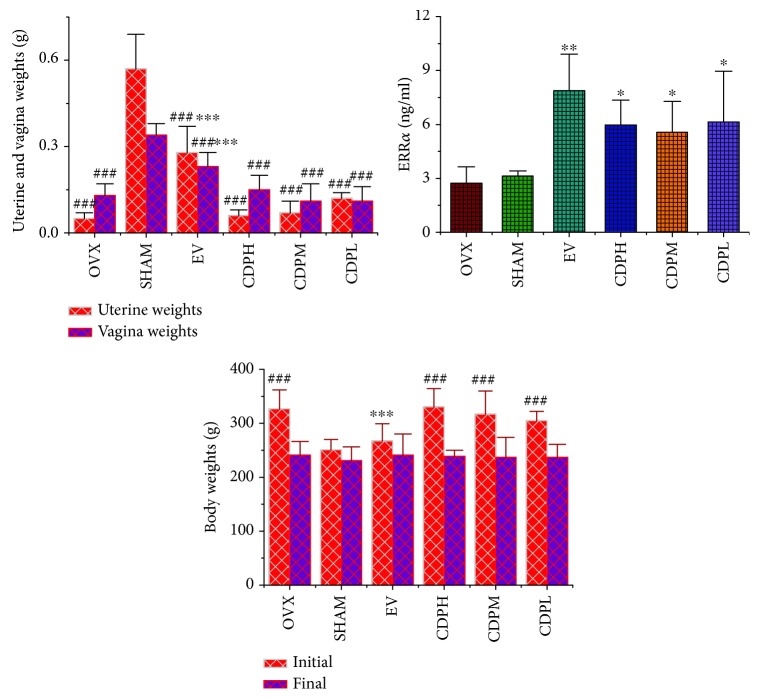
Effects of OVX and 12-week treatment with CDP or EV on ERR*α* expression, body weight, and uterine and vagina weights of rats (*n* = 10/group). Data are presented as the mean ± SD. ^∗^*p* < 0.05, ^∗∗^*p* < 0.01, and ^∗∗∗^*p* < 0.001 versus the OVX group; ^###^*p* < 0.001 versus the SHAM group.

Nonsignificant differences in the initial whole body weights of rats were observed before treatment in six groups (Figure 6). However, the ovariectomized operation led to a significant increase in the final body weight of rats in the OVX model group which is nearly 36.0%, whereas the uterine and vagina wet weights were drastically declined by nearly 90.0% and 60.0%, respectively, as compared to the SHAM rats (*p* < 0.001). Although the content of ERR*α* exhibited no significant difference between OVX and SHAM groups, all of the treatment groups including CDP and EV significantly increased the level of ERR*α*. And furthermore, when treated with EV, the above gained whole body weights as well as the loss of vagina and uterine weights of OVX rats were partly reversed (*p* < 0.001) but not affected by CDP intervention.

### 3.6. Effects of CDP on Levels of Serum MDA, SOD, and GSH

**Figure 7:**
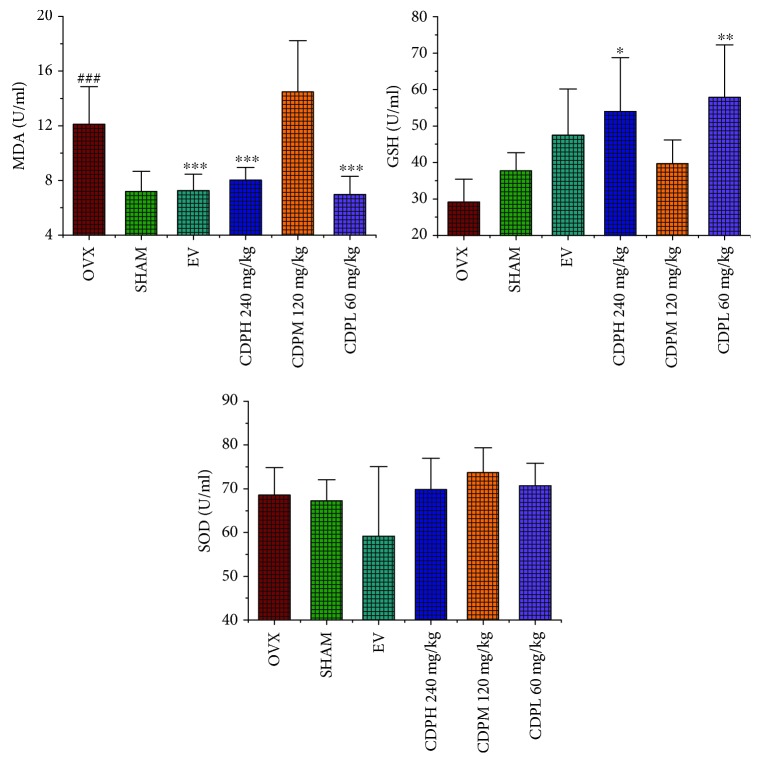
Effects of OVX and 12-week treatment with CDP or EV on serum SOD, GSH, and MDA activities of rats (*n* = 10/group). Data were described as the mean ± SD. ^∗∗^*p* < 0.01 and ^∗∗∗^*p* < 0.001 versus the OVX group; ^###^*p* < 0.001 versus the SHAM group.

There was no statistically significant difference on the properties of serum SOD and GSH between the SHAM and OVX model groups; as Figure 7 describes, an increasing trend in GSH can be observed between the above two groups. In addition, the level of serum MDA was sharply upregulated by nearly 50% in the rats of the OVX model group when compared with the SHAM rats. The activity of SOD was not influenced by CDP treatment, whereas the property of GSH was significantly improved by CDP intervention, and CDP notably decreased the level of MDA by 33.9% and 42.4% at the doses of 60 and 240 mg/kg, respectively (*p* < 0.001).

### 3.7. Effects of CDP on Protein Expression Levels

**Figure 8:**
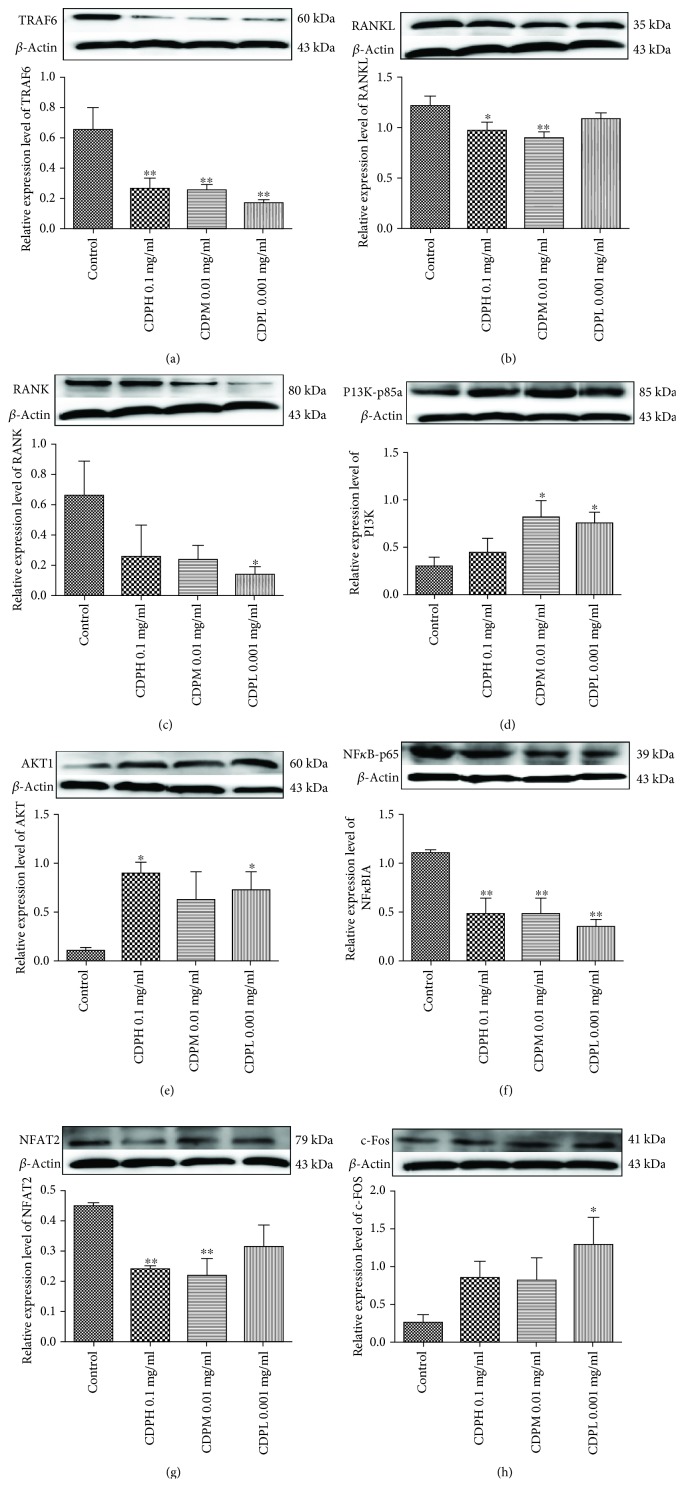
Effects of different concentrations of CDP on protein expressions of TRAF6 (a), RANKL (b), RANK (c), PI3K (d), AKT (e), NF-*κ*BIA (f), NFAT2 (g), and c-Fos (h) (*n* = 3/group); the protein expression was normalized to *β*-actin, and quantitative data of every signal protein was shown as percentages of the value of the control. Data were described as the mean ± SD. ^∗^*p* < 0.05, ^∗∗^*p* < 0.01, and ^∗∗∗^*p* < 0.001 versus the control group.

**Figure 9:**
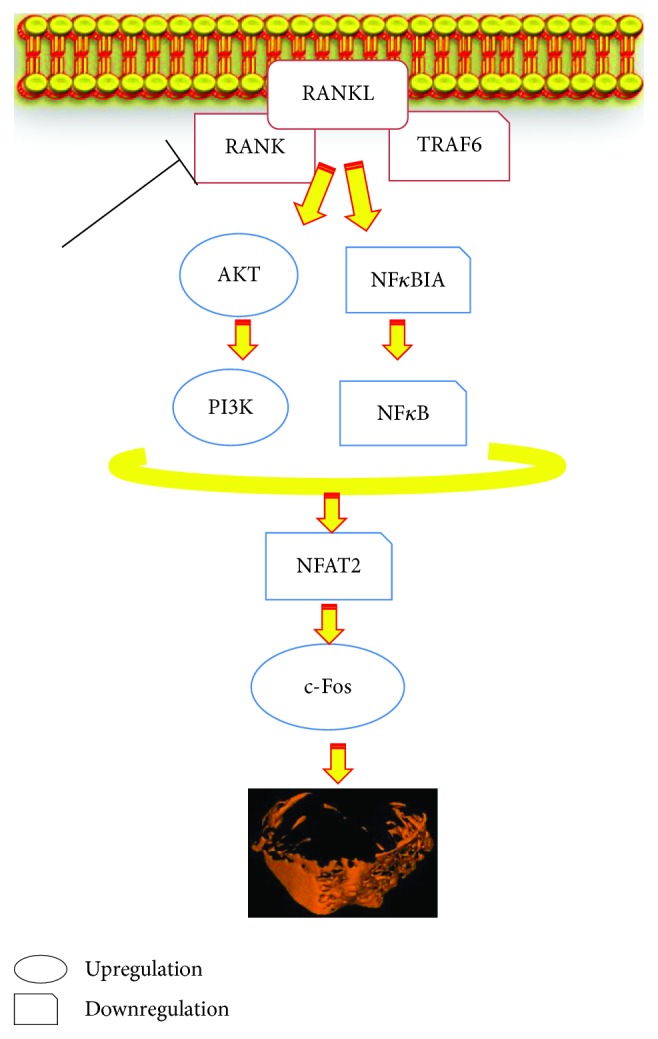
Hypothesized molecular mechanism: CDP could prevent bone loss on the OVX rat through RANKL/RANK/TRAF6-induced inactivation of NF-*κ*B and activation of PI3K/AKT pathways as well as c-Fos stimulation and NFAT2 suppression, which are evidenced by the downregulation of the expression levels of TRAF6, RANKL, RANK, NF-*κ*BIA, and NFAT2, whereas c-Fos, AKT, and PI3K were significantly upregulated by CDP treatment as compared to the control group.

Our data, shown in Figure 8, suggested that CDP treatment significantly decreased the protein levels of TRAF6, RANK, and RANKL as compared to the control. The downstream signal pathways including NF-*κ*B was suppressed, and PI3K/AKT was stimulated by CDP intervention, evidenced by the expression of NF-*κ*B-p65 downregulation, whereas PI3K-p85*α* and AKT1 were upregulated. Consequently, the expression of NFAT2 was significantly decreased, and c-Fos was obviously increased after treatment with CDP at concentration of 0.001-0.1 mg/mL. A suggested mechanism is described in Figure 9, where CDP downregulated the levels of RANKL and RANK, leading to the reduction of the binding quantities of this ligand with its receptor, and the connection of RANKL with RANK was further decreased by the downregulation of TRAF6, followed by the suppression of the downstream pathways including the NF-*κ*B pathway whereas the PI3K/AKT signal pathway was stimulated, which finally lead to the decrease of NFAT2 expression and increase of the c-Fos level.

## 4. Discussion

Phenylethanoid glycosides are naturally occurring water-soluble components which widely exist in the medicinal plant kingdom [11]. Thus far, the compounds of phenylethanoid glycosides had attracted more and more researchers because of their evident role in handling with various human aliments and abnormality [13]. Numbers of antiosteoporotic bioactive fractions and compounds, including polyphenol and phenylethanoid glycosides, were identified and isolated from dozens of natural medicinal herbs [5, 10, 12, 16, 17]. *C. deserticola* is well known as “ginseng of the desert” which implied the safety profile of this edible TCM [18, 19]. As a general tonic herb and natural health food which has long been used in Asian countries, *C*. *deserticola* exhibited beneficial function for the enhancement of kidney strength. It was found that TCM, traditionally used to invigorate and keep kidney essence, were usually used to treat osteoporosis, both *in vitro* and *in vivo* published data had proved the antiosteoporotic activity of *C*. *deserticola* [20–23], and phenylethanoid glycoside constituents including echinacoside and acteoside are the main bioactive components that exist in this edible medicinal plant; all of which suggested that not only echinacoside and acteoside themselves but also other phenylethanoid glycoside components contained in *C*. *deserticola* were considered as responsible for the antiosteoporotic property of this herb. In our present study, a favorable safety macroporous resin was used to isolate and enrich the phenylethanoid glycoside fraction from *C*. *deserticola*, and by using the HPLC method, eight main phenylethanoid glycoside components, namely, acteoside F, echinacoside, cistanoside A, acteoside, isoacteoside, acteoside C, 2′-acetylacteoside, and 6′-acetylacteoside, were found in the isolated phenylethanoid glycoside fraction, and the contents were 3.6%, 8.8%, 5.0%, 13.3%, 3.3%, 3.6%, 9.9%, and 3.2%, respectively. Echinacoside, one of the main activity compounds recorded in *C*. *deserticola* [14], had been proved possessing antiosteoporotic activity; however, the dosage of 270 mg/kg was so high which limited its further clinical application [24]. In the current experiments, the total phenylethanoid glycoside fraction with a lower dosage of 60-240 mg/kg body weight/day was used on OVX rats, and the contents of identified constituents were nearly 50% pure in this fraction by using the HPLC method.

It was well known that OVX can cause osteoporosis, and an OVX rat was believed as a classical and suitable model to simulate human postmenopausal osteoporosis. At the same time, a significant decrease in bone mineral density, trabecular bone microarchitecture, uterine and vagina wet weights, and estrogen level, as well as the obvious enhancement in bone resorption and body weight, were observed after ovariectomy surgery, of which were in part due to estrogen loss [25]. Our data, thus far, clearly demonstrated that OVX indeed induced postmenopausal osteoporosis and is always accompanied by sharp decline in bone quality, bone microarchitecture, and uterine and vagina wet weights. As EV is a general hormone replacement agent which has been used in the clinical practice, it was used as a positive control in our *in vivo* experiment, and the gained body weight and atrophy uterus weights as well as deteriorated bone mineral density and trabecular bone microarchitecture were expectedly reversed by EV supplementation. Totally different to the positive control, the decreased vagina and uterine weights as well as the gained whole body weight of rats in the OVX model group were not affected by CDP treatment, which implied that CDP could enhance the bone formation without inducing the side effects on body and uterine organic tissues. Although the levels of ERR*α* were significantly upregulated by CDP treatment, it was just like a phytoestrogen effect that no side effects on uterine and vagina organic tissues were observed. In addition, treatment of CDP significantly strengthened the quality of bone in OVX rats which had been deteriorated by ovariectomy surgery.

In addition, the levels of P and Ca in urinary and serum of OVX rats were also used to reflect the antiosteoporotic effect, and the concentrations of Ca and inorganic P were usually dependent on the levels of calcitonin and PTH [26]. In the present study, although no significant declining or increasing trends in the urinary excretion level of Ca, serum P, serum Ca, and PTH in rats of the OVX model group were obtained, the significant urinary levels of P and calcitonin (*p* < 0.001) were observed. Consistent with the published data that estrogen deficiency caused by ovariectomy surgery always led to a decreased calcitonin level in blood, this decreased serum calcitonin finally led to an increased PTH level, where Ca was believed as the major regulator of PTH secretion. Because the concentration of PTH showed no significant difference between the OVX and SHAM groups in the present study, the level of Ca in both serum and urine also exhibited no obvious changes between the above two groups. However, a significantly declined tendency on the level of calcitonin between the OVX and SHAM groups was obtained, and consequently, the content of P in urine of OVX was potently decreased. We believed that the above data may explain the contradictory phenomenon of why the urinary excretion of the Ca level in OVX rats showed no obvious change as compared to SHAM rats, and this phenomenon may be also related to the increased rate of bone turnover [27]. After treatment with CDP, the levels of P and Ca in serum were notably upregulated, and the content of P in urine was obviously downregulated in OVX rats, which reflected that CDP could not only prevent bone mineral element excretion but also enhance the serum content of those elements, thus indirectly suppressing bone loss.

Furthermore, the bone formation and resorption markers as well as the antioxidant enzymes including SOD and GSH were also employed to explain the underlying antiosteoporotic mechanisms of CDP. Similar to the published data, the level of ALP in rats of the OVX model group exhibited a nonstatistically significant increasing trend which indicate an accelerated rate of bone turnover in postmenopausal osteoporosis [10]. However, after treatment with the CDP (60, 120, and 240 mg/kg/day), the property of ALP was significantly enhanced. It was well known that OVX caused a sharp decline of estrogen levels which usually lead to an exceed bone resorption and oxidative stress [28], evidenced by the levels of TRAP, cathepsin K, and DPD as well as MDA notably upregulated in rats of the OVX model group. However, those deteriorations were partly improved by CDP intervention. In addition, OVX rat treatment with CDP (60 and 240 mg/kg) demonstrated a significant increase in activity of GSH (*p* < 0.05). The above results implied that CDP exhibited therapeutic effect on OVX-induced osteoporosis, and this effects were both by enhancing bone formation and suppressing bone resorption as well as improving the bone antioxidant system.

Activation of RANK by its ligand RANKL stimulated the expressions of NFAT2 and c-Fos via PI3K/AKT and NF-*κ*B signaling [29]. NF-*κ*B was proved essential for osteoclastogenesis as the disruption of NF-*κ*B could lead to an impaired osteoclast differentiation with an osteopetrotic phenotype, and NF-*κ*B upregulated c-Fos and downregulated NFAT2 expressions during RANKL/RANK/TRAF-induced osteoclastogenesis. To estimate the beneficial influence of CDP on NFAT2 and c-Fos-mediated osteoclastogenesis, the expression levels of RANKL and RANK were analyzed. Expectedly, CDP significantly inhibited NFAT2 and stimulated c-Fos levels through downregulating the expressions of RANKL and RANK. Meanwhile, RANK itself lacked intrinsic kinase property unless joined by TRAF6 to trigger the downstream signaling [3]. CDP also downregulated the expression of TRAF6, which led to the binding quantities of RANKL and RANK significantly reduced. A hypothesized antiosteoporotic mechanism of CDP on OVX rats covered the above signaling pathways, and regulators were described in Figure 9. Concisely, CDP declined TRAF6, RANKL, and RANK levels, thus suppressing the downstream signaling pathways including PI3K/AKT and NF-*κ*B which are triggered by RANKL/RANK, and finally reduced the expressions and activities of the key osteoclastogenic proteins NFAT2 and c-Fos. Therefore, multiple clues of data implied the beneficial effect of CDP on bone metabolism of OVX rats mainly through RANKL/RANK/TRAF6-mediated PI3K/AKT and NF-*κ*B pathways.

## 5. Conclusion

In summary, the total phenylethanoid glycosides, isolated from *C*. *deserticola*, exhibited significant beneficial effects on postmenopausal osteoporosis of OVX rats, and the therapeutic potential in suppressing bone loss was mainly through stimulating bone formation and inhibiting bone resorption as well as improving the bone antioxidant system; the mechanisms may be related to RANKL/RANK/TRAF6-induced NF-*κ*B activation and PI3K/AKT inactivation as well as c-Fos stimulation and NFAT2 suppression, and finally, the differentiation of osteoclast was inhibited.

## Data Availability

The data used to support the findings of this study are available from the corresponding author upon request.

## Abbreviations

AKT: Protein kinase B
ALP: Alkaline phosphatase
BGP: Bone gla-protein
BMC: Bone mineral content
CDP: Phenylethanoid glycoside fraction of *C. deserticola*
CK: Cathepsin K
CT: Calcitonin
DPD: Deoxypyridinoline
ERR*α*: Estrogen-related receptor alpha
EV: Estradiol valerate
GSH: Glutathione
l-PTH: lntact parathormone
MCSF: Macrophage colony-stimulating factor
MDA: Malondialdehyde
NFAT2: Nuclear factor of activated T cells c2
NF-*κ*B: Nuclear factor kappa B
OPG: Osteoprotegerin
OVX: Ovariectomized
PhGs: Phenylethanoid glycosides
PI3K: Phosphoinositide 3-kinase
RANK: Receptor activator of nuclear factor kappa B
RANKL: Receptor activator of nuclear factor kappa B ligand
ROI: Region of interest
SOD: Superoxide dismutase
Tb.N: Trabecular number
Tb.Sp: Trabecular separation
Tb.Th: Trabecular thickness
TCM: Traditional Chinese medicine
TMC: Tissue mineral content
TMD: Tissue mineral density
TNF: Tumor necrosis factor
TRAF6: Tumor necrosis factor receptor-associated factor 6
TRAP: Tartrate-resistant acid phosphatase.
